# Does PARP1 up-regulation correlate with PSMA expression in patients with metastatic castration-resistant prostate cancer studied with [^18^F]PARPi and [^68^Ga]PSMA PET/CT?

**DOI:** 10.1007/s00259-025-07448-z

**Published:** 2025-07-19

**Authors:** Holger Einspieler, Heidemarie Ofner, Marius Ozenil, Clemens P. Spielvogel, Ilva Kristiana Langrate, Melanie R. Hassler, Lukas Nics, Karsten Bamminger, Pascal A. T. Baltzer, Shahrokh F. Shariat, Marcus Hacker, Gero Kramer, Sazan Rasul

**Affiliations:** 1 Department of Biomedical Imaging and Image-guided Therapy, Division of Nuclear Medicine, Medical University of Vienna, Währinger Gürtel 18-20, Vienna, 1090 Austria; 2 Department of Urology, Comprehensive Cancer Center, Medical University of Vienna, Vienna, Austria; 3 Department of Biomedical Imaging and Image-Guided Therapy, Division of General and Pediatric Radiology, Medical University of Vienna, Vienna, 1090 Austria; 4 Department of Urology, University of Texas Southwestern Medical Center, Dallas, USA; 5 Division of Urology, Department of Special Surgery, The University of Jordan, Amman, Jordan; 6 Department of Urology, Second Faculty of Medicine, Charles University, Prague, Czech Republic; 7 Department of Urology, Weill Cornell Medical College, New York, USA; 8 Karl Landsteiner Institute of Urology and Andrology, Vienna, Austria

**Keywords:** Prostate cancer, mCRPC, PSMA, ^18^F-PARPi, PET/CT

## Abstract

**Purpose:**

[^18^F] Poly-ADP-ribose polymerase inhibitors (PARPi), a novel radiotracer, enables visualization of PARP1 upregulation by PET imaging. Here, we aimed to quantify PARPi uptake in tumor lesions of metastatic castration-resistant PCa (mCRPC) patients and perform a comparison with prostate specific membrane antigen (PSMA) expression using PET/CT scans.

**Methods:**

Data from 22 male patients with mCRPC, who underwent [^18^F]PARPi and [^68^Ga]Ga-PSMA-11 PET/CT scans, were retrospectively quantified. Lesions with relevant PARPi uptake (higher than background) were delineated and correlated with their [^68^Ga]PSMA uptake using standardized uptake values (SUV). Additionally, a comparison was performed to investigate the effects of homologous recombination deficiency (HRD) alterations on PARPi tumor uptake.

**Results:**

The majority of metastatic PCa lesions that exhibited PARPi uptake were located in the bones (*n* = 57), with mean SUVmax values of 4.9 ± 1.5 for PARPi and 30.9 ± 28.3 for [^68^Ga]PSMA. Additionally, 3 local prostate lesions, 14 lymph nodes and 4 further metastatic lesions were detected. Significant correlations were identified between PARPi- and [^68^Ga]PSMA uptake, as measured by SUVmean (*r* = 0.48, *p* < 0.001), SUVpeak (*r* = 0.48, *p* < 0.001) and SUVmax (*r* = 0.43, *p* < 0.001) of the osseous metastatic lesions and SUVpeak (*r* = 0.49, *p* = 0.04) of extraosseous lesions. No significant differences were found between PARPi uptake of metastatic lesions in patients with or without HRD alterations (all *p* > 0.05).

**Conclusion:**

Results showed a considerable uptake of [^18^F]PARPi in mCRPC patients and indicated a correlation between PARPi uptake and PSMA expression, suggesting the potential of using [^18^F]PARPi as a diagnostic imaging tool in mCRPC patients. More studies are needed to evaluate the clinical benefit of this innovative radiotracer.

## Background

Prostate cancer (PCa) is one of the most common cancers worldwide and the fifth-leading reason of cancer-related deaths in men globally [1]. The presence of distant metastatic lesions is rare at the timepoint of the initial diagnosis, nevertheless, up to 20–40% of patients suffer from a relapse of disease after primary local treatment with curative intent [2].

In metastatic hormone-sensitive PCa, androgen deprivation therapy (ADT) combined with other novel androgen receptor targeting agents remains the backbone of therapy [3–5]. Despite recent therapeutic improvements, progression to castration-resistant PCa (CRPC) remains associated with high morbidity and mortality.

At this stage, the primary tumor and its associated lesions are characterized by high expression of prostate-specific membrane antigen (PSMA), a protein that is found on the surface of highly aggressive PCa cells in 95% of patients [6]. PSMA can be radiolabeled with isotopes, making it an ideal target for diagnostic imaging and therapeutic purposes and several tracers have been approved by the regulatory authorities in the last decade [7, 8]. [^177^Lu]Lu-PSMA-617-radioligand therapies targeting the PSMA receptor were recently approved by the U.S. Food and Drug Administration (FDA) and the European Medicines Agency (EMA) and are recommended in the European Association of Urology (EAU) guidelines as a treatment option for metastatic CRPC (mCRPC) patients [9]. [^68^Ga]PSMA PET is a widely used imaging modality, providing high sensitivity and specificity in detecting PCa lesions, even in recurrent disease with low measured PSA levels [10]. Especially in the detection of lymph node metastasis, PSMA PET/CT has been shown to have a significantly higher sensitivity and specificity compared to conventional imaging (e.g. sensitivity 85% vs. 38%, specificity 98% vs. 91%, respectively) [10–12]. Due to its valuable diagnostic characteristics, [^68^Ga]PSMA PET is not only an established imaging modality in the clinical routine in the initial diagnostic work-up of high risk localized PCa, biochemical recurrence or as a follow-up staging modality in the metastatic setting, but is also used as a comparator arm in various imaging-based clinical trials [13–17].

Given the continuous emergence of new therapeutic agents in PCa, the ability to visualize relevant molecular targets through PET imaging is a promising way to help guide clinical decision making and patient selection.

A major advancement in recent years was the identification of germline and somatic alterations in a significant number of PCa patients, e.g. mutations in genes associated with the homologous recombination repair (HRR) pathway, e.g. BRCA1, BRCA2 and ATM, in approximately 25% of patients with mCRPC [18]. Patients with such mutations, harboring homologous repair deficiencies (HRD), which refers to the functional state of an altered repair mechanisms of DNA double-strand breaks, reveal an aggressive disease pattern and are at higher risk of poor survival outcomes [19]. However, these mutations also present a distinct opportunity to design therapeutic approaches that exploit the reduced capacity of tumor cells to repair DNA damage [20]. Most recently, a novel therapeutic approach, poly ADP-ribose polymerase (PARP) inhibitors, have been developed. PARP inhibitors are targeted therapies that disrupt the DNA repair function of PARPs, demonstrating synthetic lethality [21]. The administration of PARP inhibitors has already been examined in phase III clinical trials (e.g. PROFOUND, PROpel, TALAPRO 3), demonstrating impressive results as therapeutic option in mCRPC patients [22, 23]. These recently published clinical trials reinforce the need to identify patients who may benefit most from PARP inhibitor therapy, which could have a huge impact on clinical routine.

The question of whether patients with HRR pathway alterations derive increased therapeutic benefit from PARP inhibitors remains unanswered, as the inclusion criteria in published trials vary, with some studies including all patients and others focusing specifically on those with HRR pathway alterations. For example, TALAPRO 3, which investigated talazoparib in combination with enzalutamide, showed a significant therapeutic response also for patients without HRR gene alterations [24].

In this regard, several novel radiotracers such as [^18^F]PARPi and [^18^F]FluorThanatrace ([^18^F]FTT), derived from olaparib and rucaparib, respectively, have recently been developed, enabling the visualization of PARP1 up-regulation through PET imaging [25–28]. [^18^F]PARPi might therefore be an appropriate and promising in vivo radioligand, to detect PARP-expressing lesions. To our knowledge, no [^18^F]PARPi imaging results have yet been published in PCa patients.

Since therapies targeting both PSMA and PARP1 pathways are now available for mCRPC, we sought in this study to investigate the relationship between [^68^Ga]PSMA and [^18^F]PARPi uptake by detecting and quantifying PARPi versus [^68^Ga]PSMA uptake in different tumor lesions in mCRPC patients using PET/CT scans.

## Methods

### Patients

This retrospective analysis included data from 22 PARP inhibitor-naïve patients with mCRPC. All patients underwent both [^18^F]PARPi PET/CT and [^68^Ga]Ga-PSMA-11 PET/CT scans within 1 month between March 2024 and November 2024. [^68^Ga]Ga-PSMA-11 PET/CT scan was performed as part of the routine clinical staging of the PCa and PARPi-PET/CT examination was done as part of the “named patient program”. This retrospective analysis received ethical approval by the ethics committee of the Medical University of Vienna (1745/2021) and all patients gave their written consent prior to the examinations.

Moreover, genetic and somatic testing were conducted in some patients as part of the clinical routine, adhering to international guidelines and performed after obtaining their informed consent in compliance with Austrian genetic testing regulations. The panels used for the genetic testing of the included patients were: Hereditary Cancer Solution Panel (HCS v1.1 CE-IVD; SOPHiA Genetics) for germline testing, AmoyDx Comprehensive Panel (AmoyDx) for the identification of somatic mutations from liquid biopsies, and TruSight Oncology 500 Assay (Illumina) for somatic testing. DNA for germline or somatic testing through liquid biopsy was isolated from blood, libraries of the amplified target DNA were prepared, and sequencing was performed on a MiSeq or NextSeq500 instrument (Illumina).

### PET/CT Examinations

All PET/CT scans were acquired with a Siemens Biograph 128 Vision Quadra Edge. In every examination, whole body scans from the skull to the mid-thigh were obtained 45–60 min after the injection of 3 MBq/kg body weight [^18^F]PARPi (synonymous with [^18^F]ATD001) or 150 ± 10 MBq [^68^Ga]Ga-PSMA-11.

The CT acquisition protocols for [^18^F]PARPi and [^68^Ga]Ga-PSMA-11 PET scans were consistent within each radiopharmaceutical type, following the routine clinical practice at our institution: For [18F]PARPi PET/CT, low-dose CT scans were acquired between 80 and 120 kV and 30–40 mAs. When the [68Ga]Ga-PSMA-11 PET/CT was performed, diagnostic contrast-enhanced CT scans were conducted between 80 and 140 kV and 80–180 mAs. A CT matrix of 512 × 512 and slice thickness of 2 mm were used in all scans. [^18^F]PARPi PET and [^68^Ga]Ga-PSMA-11 PET examinations were performed with a duration of 2.5 and 4.5 min, respectively, in one position with a field of view (FOV) of 106 cm employing an iterative reconstruction technique that utilized a point-spread-function-based algorithm. Subsequent to this, corrections for scatter and attenuation were applied based on the CT scan (PET matrix sizes: 440 × 440 and 220 × 220, respectively).

### Image analysis

The image analysis was conducted on a workstation utilizing Hybrid 3D software (version 4.17, Hermes Medical Solutions, Stockholm, Sweden). The PET image intensities (Bq/mL) were transformed into standardized uptake values (SUV) normalized to body weight. The collection of SUVmean, SUVpeak and SUVmax (maximum) values for the organs and lesions was conducted by two experienced nuclear medicine physicians. Firstly, lesions with higher PARPi uptake than the background were delineated in [^18^F]PARPi PET/CT and afterwards in [^68^Ga]Ga-PSMA-11 PET/CT. Secondly, cuboidal volumes of interest (VOIs) were manually placed in non-tumor organs, with a target size of 13.50 mL in the liver, in the bone marrow and in the gluteus maximus muscle and a target size of 1.60 mL in the abdominal aorta, avoiding the inclusion of spill-in activity or metastatic lesions.

### Radiotracer synthesis

[^18^F]PARPi was synthesized in an automated Elixys Radiosynthesis Module (SOFIE) by adapting a literature reported procedure [29].

In short, 4-[^18^F]fluorobenzoic acid was synthesized by reacting dry [^18^F]fluoride with ethyl-4-nitrobenzoate, followed by aqueous hydrolysis with tetrabutylammonium hydroxide. Subsequent amide coupling with an amine precursor yielded [^18^F]PARPi, which was purified by semi-preparative HPLC and solid phase extraction before physiological formulation and sterile filtration. The radiosynthesis provided > 0.98 GBq [^18^F]PARPi per batch, with radiochemical purity > 99%.

[^68^Ga]Ga-PSMA-11 was synthesized using the LOCAMETZ kit (Novartis Pharma GmbH) and a GalliAd radionuclide generator (IRE ELiT), following the prescribed protocols outlined in the product information.

### Statistical analysis

Descriptive variables were summarized as mean (± standard deviation (SD)) for normally distributed data and as median (minimum; maximum) for non-normally distributed data. Categorical variables were shown as frequencies and percentages. The t-test was used to compare continuous parameters of two groups if normally distributed and the Mann–Whitney U test was used to compare continuous outcomes if non-normal. Correlation tests were performed using the Pearson correlation test if the underlying data was normally distributed or a Spearman rank correlation test if not normally distributed. Necessity for parametric assessments was performed using the Shapiro Wilks test. A p-value of < 0.05 was determined to be statistically significant. All p-values are to be interpreted exploratorily. Missing data were addressed using pairwise deletion in this retrospective analysis, excluding only cases with missing values for the relevant variables. In addition, graphical representations included box plots to illustrate different SUV values and a regression plot with 95% confidence intervals to demonstrate the relationship between pairs of continuous parameters. All statistical analysis were performed with either IBM Mac SPSS Version 28 or Python 3.9.5 and the packages SciPy [30] and Stat annotations.

## Results

### Patients

Clinical and imaging data of 22 PARP inhibitor-naïve men with advanced PCa were retrospectively analyzed. Demographic characteristics of this investigated cohort are illustrated in Table 1.

**Table 1:** Demographic parameters of all studied patients:

Clinical Parameters	All Patients
Patients– n	22
Age in years - Mean ± SD	70.5 ± 7.7
BMI - mean ± SD	26.5 ± 4.0
PSA (at scan) ng/mL - Median (max.; min.)	43.25 (4409; 0.19)
HRR pathway mutations– n (%) - germline - somatic	6 (27) 3 (13.6) 3 (13.6)
Previous treatments: RPE - n (%) EBRT - n (%) Hormone therapy - n (%) - ADT - n (%) - ARTA - n (%) Chemotherapy - n (%) - Docetaxel- n (%) - Cabazitaxel- n (%) PSMA radioligand therapy - n (%)	12 (55) 4 (18) 22 (100) 16 (73) 19 (86) 18 (82) 18 (82) 8 (36) 12 (55)

*n* number of patients, *SD* standard deviation, *BMI* body mass index, *PSA* prostate specific antigen, *max* maximum, *min* minimum, *HRR* homologous recombination repair, *RPE* radical prostatectomy, *EBRT* external beam radiation therapy, *ADT* androgen deprivation therapy, *ARTA* androgen receptor-targeted agents

### PET parameters

The mean SUVmean, SUVpeak and SUVmax values of healthy organ tissues as well as of all metastatic and local prostate tumor lesions of all patients, extracted from PARPi- and [^68^Ga]Ga-PSMA-11 PET/CT scans, are illustrated in Table 2.

**Table 2:** PET parameters of the physiological uptake of organs as well as of all metastatic and local prostate tumor lesions of the investigated patients

Organ/lesions	[^18^F]PARPi SUVmean	[^18^F]PARPi SUVpeak	[^18^F]PARPi SUVmax	[^68^Ga]PSMA SUVmean	[^68^Ga]PSMA SUVpeak	[^68^Ga]PSMA SUVmax
Non-Tumor Organ Uptake Liver Bone marrow Muscle Abdominal Aorta	4.9 (± 1.1) 0.4 (± 0.2) 0.7 (± 0.2) 1.4 (± 0.4)	5.6 (± 1.4) 0.7 (± 0.2) 0.9 (± 0.2) 1.6 (± 0.5)	6.6 (± 2.1) 0.8 (± 0.4) 1.3 (± 0.3) 2.0 (± 0.7)	5.2 (± 2.1) 0.9 (± 0.7) 0.6 (± 0.2) 1.9 (± 0.6)	6.0 (± 2.2) 1.6 (± 1.4) 1.0 (± 0.3) 2.2 (± 0.8)	6.9 (± 2.5) 1.9 (± 1.7) 1.1 (± 0.4) 2.6 (± 0.9)
All metastatic LN (*n* = 14) Supradiaphragmatic (*n* = 8) Infradiaphragmatic (*n* = 6)	*3.5* (*± 0.7)* *3.7* (*± 0.5)* *3.3* (*± 0.8)*	*2.9* (*± 0.4)* *3.0* (*± 0.5)* *2.7* (*± 0.9)*	*5.2* (*± 1.3)* *5.3* (*± 1.0)* *4.9* (*± 1.8)*	*14.2* (*± 11.5)* *15.4* (*± 13.4)* *12.2* (*± 7.8)*	*20.1* (*± 30.3)* *26.4* (*± 37.9)* *9.9* (*± 5.3)*	*37.3* (*± 55.2)* *48.4* (*± 69.0)* *19.1* (*± 11.3)*
All metastatic bone lesions (*n* = 57) Shoulder (*n* = 5) Pelvis (*n* = 17) Upper and lower limbs (*n* = 8) Vertebrae (*n* = 14) Sternum (*n* = 4) Ribs (*n* = 9)	*3.5* (*± 1.0)* *3.1* (*± 0.4)* *2.8* (*± 1.0)* *3.7* (*± 0.5)* *4.5* (*± 0.7)* *3.6* (*± 0.6)* *3.3* (*± 0.7)*	*3.5* (*± 1.0)* *2.5* (*± 0.4)* *2.4* (*± 0.8)* *3.2* (*± 1.2)* *4.2* (*± 1.0)* *3.4* (*± 0.6)* *2.7* (*± 1.0)*	*4.9* (*± 1.5)* *4.0* (*± 0.6)* *3.8* (*± 1.2)* *5.7* (*± 1.6)* *6.3*(*± 1.1)* *5.7* (*± 0.9)* *4.7* (*± 1.7)*	*13.9* (*± 9.2)* *7.8* (*± 8.2)* *9.6* (*± 6.8)* *21.2* (*± 10.8)* *18.4* (*± 10.1)* *18.5* (*± 8.6)* *13.5* (*± 5.8)*	*16.7* (*± 14.6)* *7.6* (*± 9.6)* *9.9* (*± 7.7)* *27.3* (*± 19.9)* *22.6* (*± 18.6)* *20.0* (*± 8.8)* *17.5* (*± 13.0)*	*30.9* (*± 28.3)* *12.4* (*± 14.1)* *17.0* (*± 11.6)* *55.9* (*± 41.3)* *43.0* (*± 30.5)* *30.1* (*± 15.5)* *39.6* (*± 37.9)*
Prostate lesions (*n* = 3)	*6.2* (*± 3.3)*	*5.4* (*± 3.4)*	*17.6* (*± 15.0)*	*7.4* (*± 8.9)*	*8.8* (*± 10.4)*	*13.0* (*± 16.9)*
Other organ lesions* (*n* = 4)	*3.1* (*± 1.3)*	*3.0* (*± 1.9)*	*4.8* (*± 2.5)*	*13.1* (*± 16.4)*	*18.6* (*± 23.4)*	*33.6* (*± 40.5)*

*LN *lymph nodes, *n* number of lesions, *: including metastatic lesions in rectum and lung

The majority of metastatic PCa lesions demonstrating PARPi uptake were located in the bones (*n* = 57), with the pelvis being the most frequently affected region (*n* = 17), followed by the vertebrae (*n* = 14). Additionally, 3 local prostate tumor lesions and 18 extraosseous metastatic lesions, including 14 metastatic lymph nodes and 4 further lesions (rectum lesion (*n* = 3) and lung (*n* = 1)), were detected.

### HRD (homologous recombination deficiency) mutations

Of the 22 mCRPC men included in this study, 6 patients were diagnosed with one HRR pathway mutation, consisting of 5 patients with BRCA and 1 patient with ATM mutations. 2 patients were diagnosed with a germline BRCA2 mutation and 1 patient with a germline ATM mutation. In 3 other patients, a somatic BRCA2 mutation was detected in ctDNA through a liquid biopsy.

**Fig. 1:**
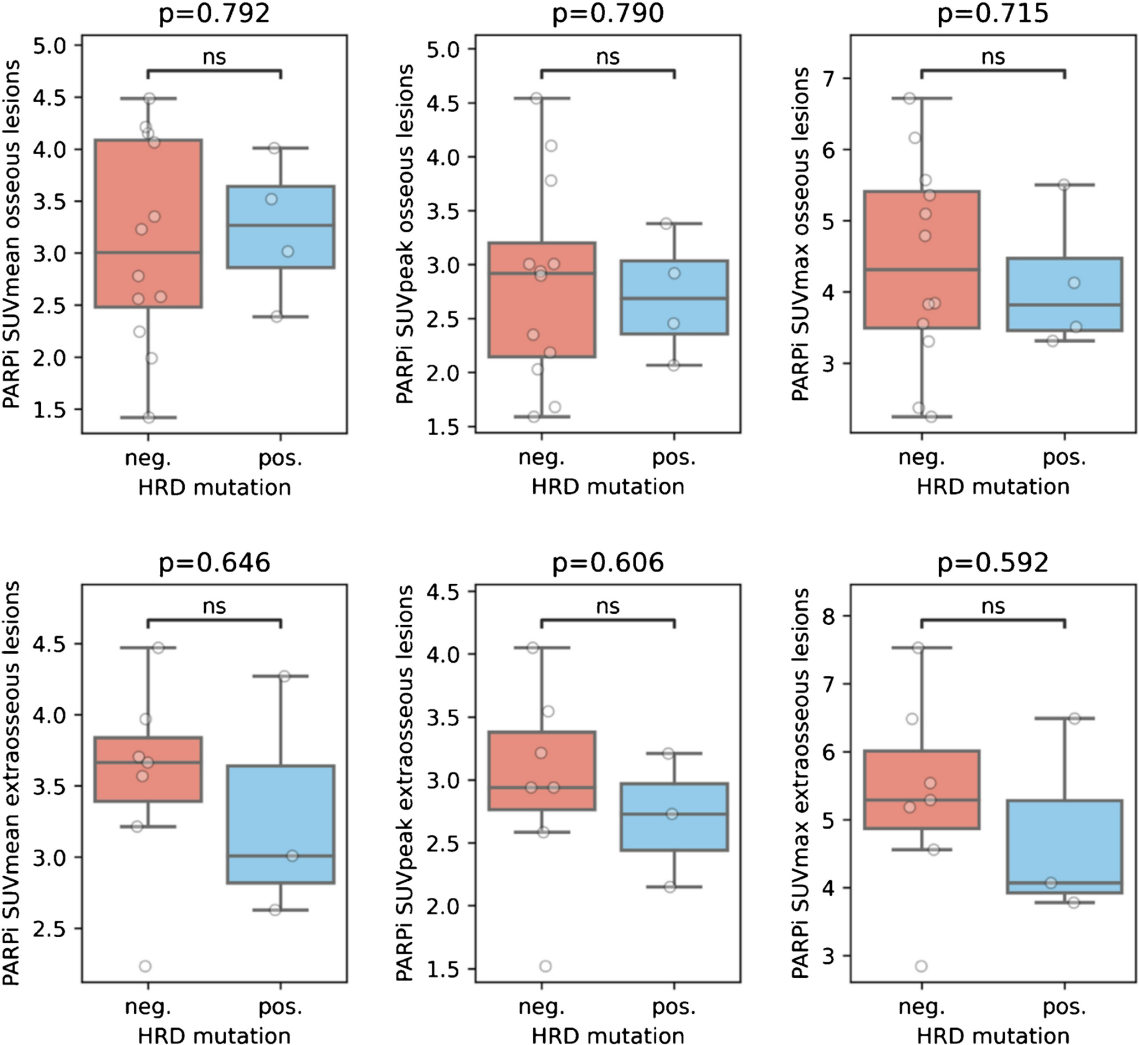
[^18^F]PARPi uptake in osseous and extraosseous metastatic lesions in relation to the HRD mutation

**Fig. 2:**
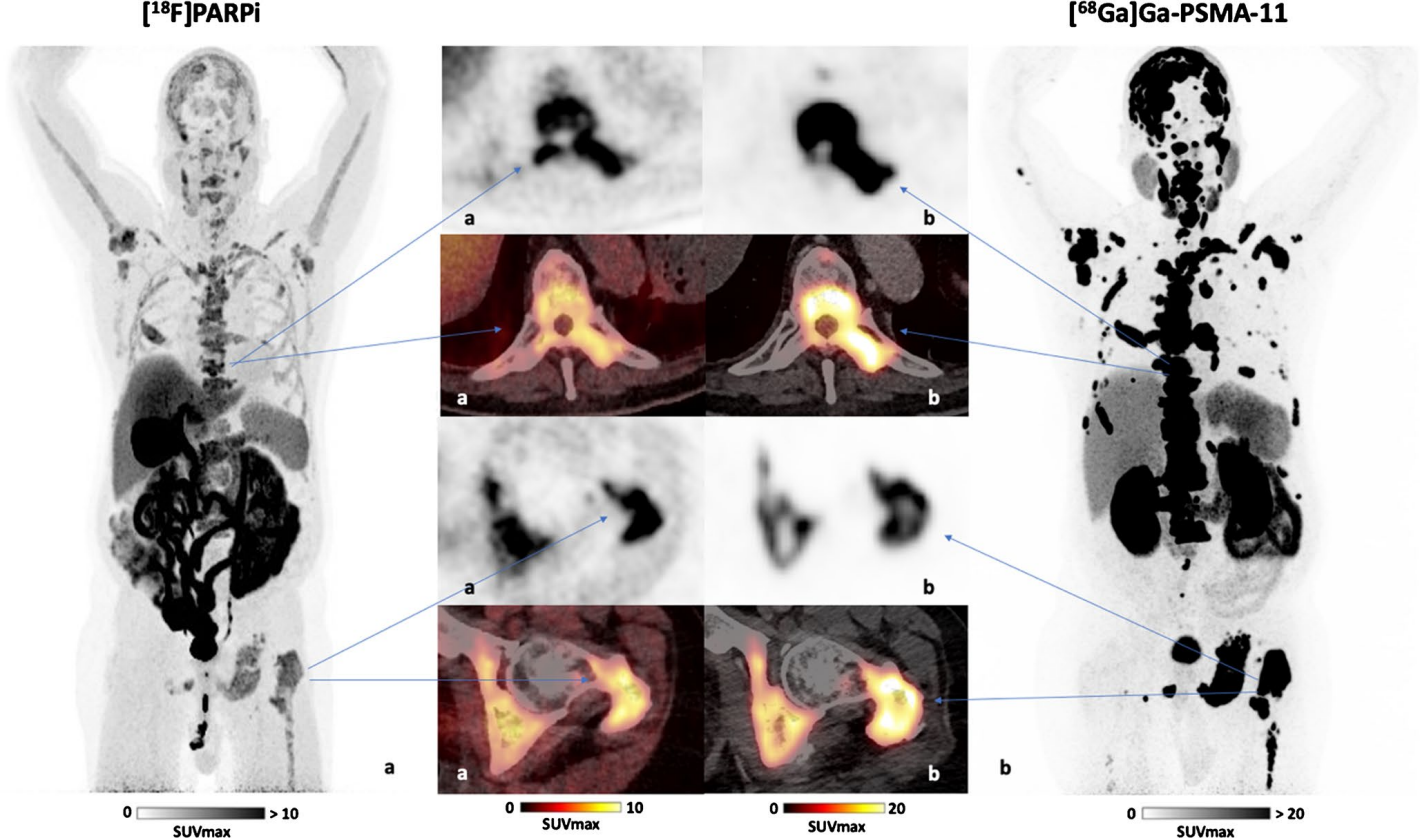
Maximum intensity projection (MIP) PET images of patient A: PET/CT images of a 63-year-old patient with metastatic castration-resistant prostate cancer (mCRPC) without a proven HRD mutation having a PSA value of 2199 ng/ml at the time of imaging. Multiple bone lesions were detected using (**a**) [^18^F]PARPi PET/CT, with a maximum standardized uptake value (SUV_max_) of 6.8, and (**b**) [^68^Ga]Ga-PSMA-11 PET/CT, with a maximum SUV_max_ >50.0

**Fig. 3:**
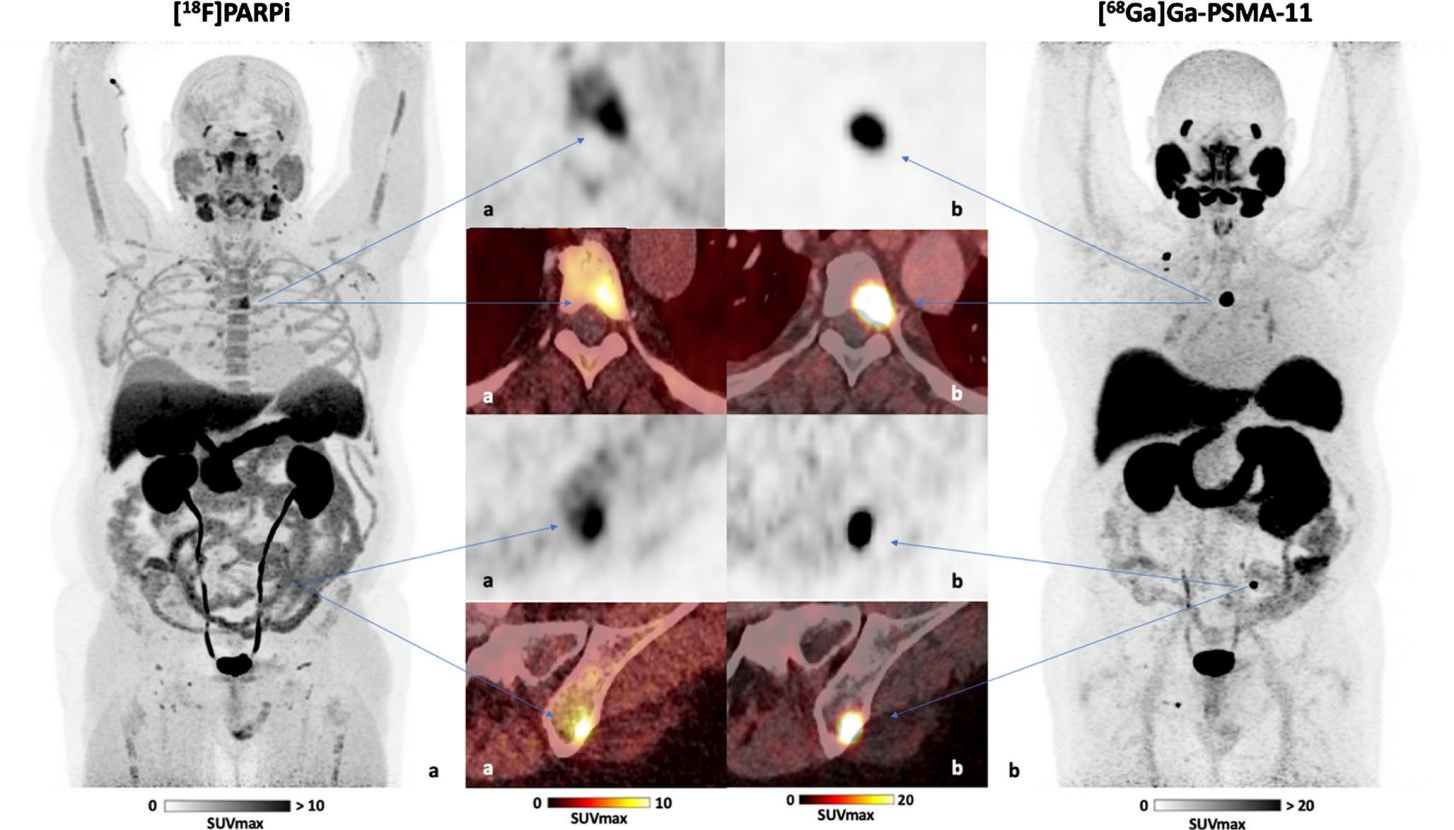
Maximum intensity projection (MIP) PET images of patient B: PET/CT images of a 55-year-old patient with metastatic castration-resistant prostate cancer (mCRPC) who has a confirmed BRCA2 mutation (HRD-positive) and a PSA level of 0.2 ng/ml at the time of imaging. Some bone metastases were identified using (**a**) [^18^F]PARPi PET/CT, showing a maximum standardized uptake value (SUV_max_) of 6.5, and (**b**) [^68^Ga]Ga-PSMA-11 PET/CT, with an SUV_max_ >50.0

Among them, patients with HRD showed significantly lower PSA values prior to the [^18^F]PARPi scans (*p* = 0.046) than patients without HRR pathway mutations (PSA: 30.75ng/mL versus 832.73ng/mL; respectively). No significant differences were observed in patients with and without HRR pathway mutations comparing SUVmean (3.2 ± 0.7 vs. 3.1 ± 1.0), SUVpeak (2.7 ± 0.6 vs. 2.8 ± 0.9), and SUVmax (4.1 ± 1.0 vs. 4.4 ± 1.4) of osseous lesions (all *p* > 0.05) and SUVmean (3.3 ± 0.9 vs. 3.5 ± 0.7), SUVpeak (2.7 ± 0.5 vs. 3.0 ± 0.8), and SUVmax (4.8 ± 1.5 vs. 5.3 ± 1.5) of extraosseous metastatic lesions (all *p* > 0.05), Fig. 1. Examples of patients with and without HRR pathway mutation can be seen in Figs. 2 and 3.

### Correlation of [^18^F]PARPi and [^68^Ga]Ga-PSMA-11 uptake

**Fig. 4:**
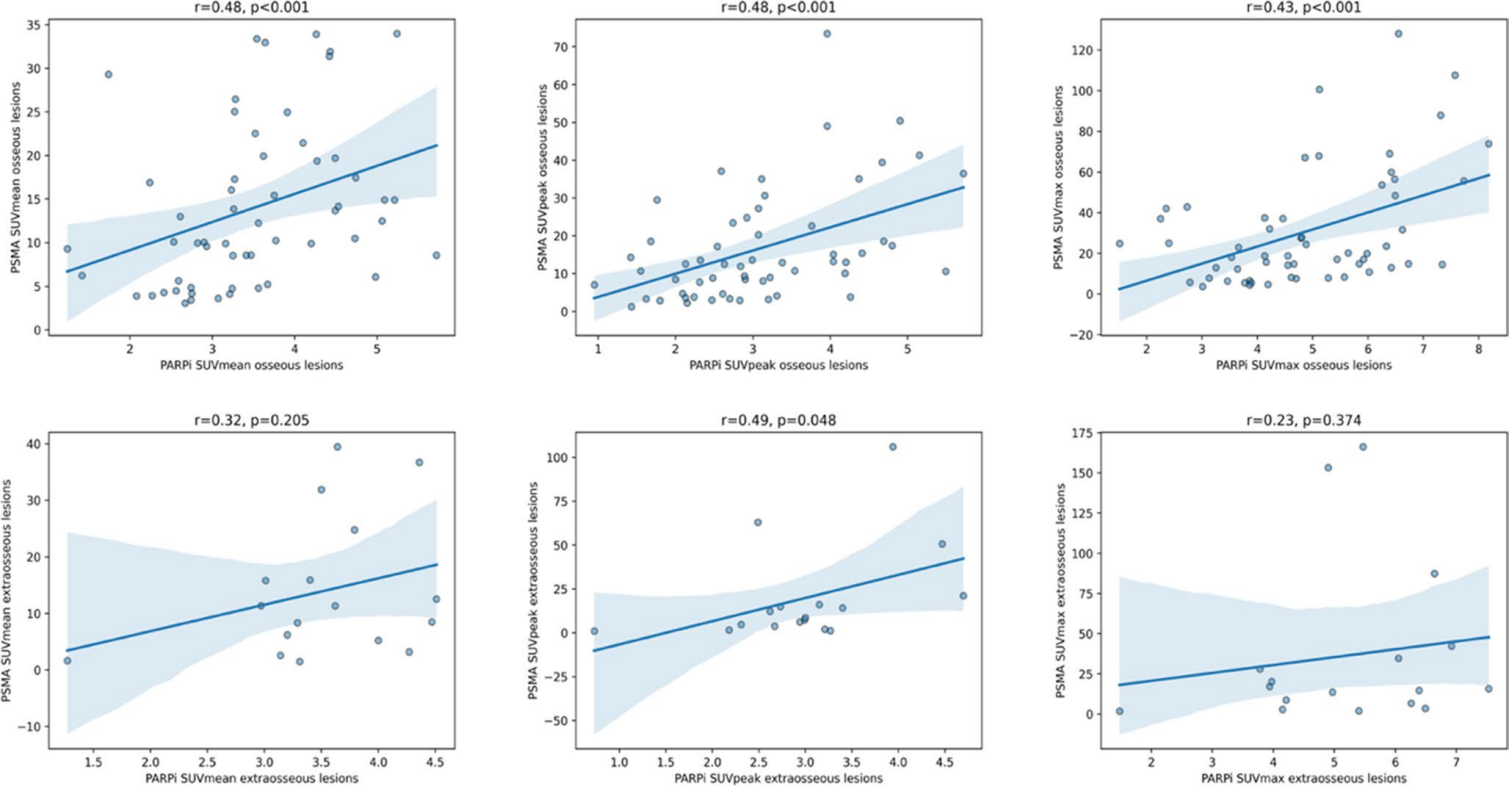
Scatterplots demonstrating correlations between [^18^F]PARPi- and [^68^Ga]Ga-PSMA-11 uptake of all osseous and extraosseous metastatic lesions

Examining PARPi- and [^68^Ga]PSMA uptake of healthy organs and metastases, significant weak to moderate correlations were identified between the uptake of both tracers, as measured by the SUVmean (*r* = 0.47, *p* = 0.028) and SUVmax (*r* = 0.45, *p* = 0.034) of the muscle, SUVmean (*r* = 0.44, *p* = 0.043) of the bone marrow, SUVpeak (*r* = 0.49, *p* = 0.048) of the extraosseous metastatic lesions and SUVmean (*r* = 0.48, *p* < 0.001), SUVpeak (*r* = 0.48, *p* < 0.001) and SUVmax (*r* = 0.43, *p* < 0.001) of the osseous metastatic lesions, Fig. 4. Weak to moderate, but not statistically significant, correlations were observed for SUVmean (*r* = 0.32) and SUVmax (*r* = 0.23, both *p* > 0.05) in extraosseous metastatic lesions when comparing both tracers.

### Correlation between [^18^F]PARPi uptake and PSA values

No significant correlations were found between patient-based PARPi uptake in osseous- and extraosseous metastatic lesions and PSA values (all *p* > 0.05).

### Observations of patients undergoing PARP inhibitor therapy following initial [^18^F]PARPi imaging

Two patients received PARP inhibitor therapy with Olaparib (LYNPARZA^®^) within three weeks after the PARPi PET scan, along with follow-up PSA values (Figs. 2 and 3). Follow-up PSA data revealed significant reductions in PSA levels following treatment: In patient A (Fig. 2), PSA decreased by 97% after 3 months of PARP inhibitor therapy. Similarly, patient B (Fig. 3) showed a 90% reduction in PSA after 3.5 months of treatment.

## Discussion

This retrospective analysis investigates the use of [^18^F]PARPi PET imaging in mCRPC patients, treatment-naïve to PARP inhibitors, comparing its expression pattern to [^68^Ga]PSMA PET. While [^18^F]PARPi PET scan has been described in patients with small cell lung cancer, head and neck cancers as well as glioblastoma, this study is, to our knowledge, the first study investigating this imaging modality in PCa patients [26, 31, 32].

Indeed, deeper understanding of the biology of PCa has been gained through investigating different molecular changes throughout the natural history of PCa and identifying the high prevalence of somatic and/or germline HRR mutations, e.g. alterations in BRCA1, BRCA2 and ATM genes. Several PARP inhibitors have gained approval in Europe, with authorizations differing concerning the use in all-comer patients or in HRR pathway mutated patients only, as well as in the concomitant treatment with an ARTA, which might lead to a synergistic effect of this treatment combination [22, 33, 34]. PARP1, a member of the PARP enzymes family, is known for its ability to recognize but also repair especially single-strand DNA breaks [35]. With the inhibition of PARP1, the accumulation of single-strand breaks can lead to an increase in double-strand DNA breaks, ultimately resulting in apoptosis [36]. Homologous recombination (HR) plays an important role in the repair of double-strand DNA breaks and patients harboring HRR pathway alterations are therefore known to be prone to genomic instability, a key hallmark of cancer development and growth. Nevertheless, this state has not only been associated to HRR altered patients [37]. Recently, the PARP inhibitor-vulnerable state described as ‘BRCAness’ was also found in non-HRR altered cells, e.g. caused by treatment with ARTAs. Five genes critically involved in HR were found to be suppressed in CRPC cells under enzalutamide, therefore suggesting a pharmaceutically induced ‘BRCAness’, potentially providing a biological rationale for the expanded use of PARP inhibitors, especially in the combination with ARTAs [38]. With the available clinical outcome data on the effectiveness of PARP inhibitors in non-HRR altered cohorts, as provided by e.g. TALAPRO 3, the expression of PARP1 as a potentially HRR-independent predictive biomarker is currently under investigation of ongoing trials [39].

In this study, we observed no significant differences in [^18^F]PARPi PET uptake between patients with or without HRR pathway mutations, highlighting the need for gaining a better understanding in the therapeutic role of PARP inhibitors in all-comer patients without HRR pathway alterations. While germline mutations are present in every cell of the body, somatic tumor mutations arise in tumor development and are specific to these cells. [^18^F]PARPi PET uptake was not associated to HRR mutations in our patients, which might provide an additional rationale for PARP1 as a therapeutic target in all-comer patients and strengthens the results of published phase III trials, showing a statistically significant therapeutic benefit in these patients [24, 40]. Additionally, no association between PSA and [^18^F]PARPi PET uptake was found, underscoring the independence of PARP1-expression compared to conventionally used biomarkers such as serum PSA. Nevertheless, the results of our study are hypothesis generating with a small, included sample size with only 27% of patients harboring either a somatic or germline alteration, which can be a confounding factor to our results. Of future interest could be to investigate the PARP1 expression in tumor tissue of patients treated with PARP inhibitors to further understand the biological mechanisms of response but also resistance, and potentially correlate it with our findings from [^18^F]PARPi-imaging.

Our results suggest a relationship between [^18^F]PARPi- and [^68^Ga]PSMA PET uptake in metastatic lesions in mCRPC patients. The majority of PARPi-expressing lesions in our patient cohort were bone lesions, and a significant yet moderate correlation between both tracers was observed regarding values of SUVmean, SUVpeak and SUVmax. One plausible explanation is that both PARP1 and PSMA are independently associated with aggressive tumor phenotypes and advanced stages of disease. As prostate cancer becomes more aggressive, it may accumulate DNA repair defects or genomic instability, increasing reliance on PARP1. Simultaneously, adaptation to androgen-deprived conditions may upregulate PSMA expression, ultimately leading to co-expression of both markers [41, 42]. However, the exact biological mechanism underlying the observed significant correlation between [^18^F]PARPi and [^68^Ga]PSMA uptake, particularly in bone metastases but only partially in extraosseous lesions, remains unclear. Another possible reason is the smaller number of extraosseous lesions compared to osseous sites (*n* = 21 vs. *n* = 57, respectively), which may limit statistical power. Additionally, partial volume effects (PVE) may contribute to discrepancies in uptake measurements across lesion types, as smaller lesions such as lymph nodes are more susceptible to underestimation of tracer uptake due to limited spatial resolution [43]. This is further supported by our observation, as shown in Table 2, that SUVpeak values were at times paradoxically lower than SUVmean values, a known phenomenon associated with PVE-related distortion of uptake quantification.

Nevertheless, the unique characteristics of bone microenvironment may influence tumor behavior in ways that do not occur in soft tissues and the mechanism, by which PCa often creates distant metastasis prominently to the bone, are still under investigation. The bone marrow is known to have a microenvironment with low oxygen tension in general, and hypoxia is also a significant hallmark of tumor microenvironment [44, 45]. Hypoxia, commonly observed in poorly vascularized bone lesions, may upregulate PSMA as an adaptive mechanism, while potentially result in increased PARP activities, suggesting the activation of DNA repair mechanisms [46–48].

Furthermore, the tumor mutational profile is under current investigation of different trials and preliminary data showed a distinct tumor mutational profile depending on the site of metastasis. Besides the biological aspect, this has also been shown to impact survival outcomes of patients, with the anatomical site of metastasis formation being a prognostic factors of disease [49, 50]. These different possible explanations only highlight the need for larger, site-specific analyses in future studies.

While other studies have evaluated PARP1 expression with different PET tracers, this is, to our knowledge, also the first study, directly comparing PARP1 expression through [^18^F]PARPi uptake with [^68^Ga]PSMA PET uptake. Besides the practical impact on clinical routine with PSMA PET/CT being an established staging modality, the comparison between the two tracers also underlines the potential biological link between androgen receptor dependent-pathways and PARP1 inhibition, as for example in inducing a pharmaceutical ‘BRCAness’. The ability of assessing two possible treatment options with a non-invasive approach through PET imaging may be used as a tool for clinical patient selection in the future.

While several studies have already demonstrated a correlation between the uptake of PARP imaging agents and elevated intratumoral PARP1 expression, the clinical utility of [^18^F]PARPi imaging in PCa patients has yet to be fully elucidated [25, 51]. Our findings of high [^18^F]PARPi uptake in metastatic PCa lesions support these observations and underscore the need for further investigation in this tumor entity. Interestingly, a closely related tracer to [^18^F]PARPi, [^18^F]FTT, has already shown a link between higher pre-treatment tracer uptake and better therapeutic outcomes for breast cancer patients receiving PARP inhibitor therapy [52]. Furthermore, although it was not the primary focus of this study, we observed promising results in our own cohort. Two patients with metastatic lesions showing [^18^F]PARPi uptake received PARP inhibitor therapy following [^18^F]PARPi imaging. Both patients demonstrated a significant PSA decline, with reductions of ≥ 90%. These findings collectively suggest that [^18^F]PARPi imaging may be valuable not only for identifying patients with elevated PARP1 expression but also for guiding personalized treatment strategies in metastatic prostate cancer.

A major limitation of this study is its retrospective nature, thereby being prone to biases and confounders. Additionally, the small sample size in this study diminishes the statistical power and might lead to a reduced generalizability of our results. The high heterogeneity of the included patients concerning the pre-treatments, stage and volume of disease and localization of metastasis is also to be noted, even though a high heterogeneity of disease is a common feature in metastatic PCa patients and a unique factor of the disease. The heterogeneity of our cohort is further exemplified by the wide interquartile range of PSA values observed. Although PSA is commonly used as a surrogate biomarker for tumor burden and disease aggressiveness in PCa, we unexpectedly observed significantly lower PSA levels in patients with HRR pathway alterations compared to those without. While PSA levels are not directly associated with the presence of HRR mutations, this finding may be influenced by confounding factors, including the limited sample size, variability in tumor volume, and differences in prior treatments among patients. These factors likely contributed to the observed discrepancies and underscore the need for cautious interpretation of the results. Somatic testing in the included patients was conducted via liquid biopsies, testing cell-free tumor DNA in blood plasma. Thereby, somatic mutations cannot be linked to a specific metastasis location and the exact location of mutated tumor cells cannot be identified, which might have an impact on [^18^F]PARPi uptake. Nevertheless, somatic HRR mutations in PCa have been described to take place in early PCa development with a high prevalence already in mHSPC and somatic HRR mutations might be present in most metastatic sites [53].

This study demonstrates the feasibility of [¹⁸F]PARPi PET imaging in mCRPC might provide the base of a rationale for combined targeted therapies involving PARP inhibitor and PSMA-directed approaches, but its clinical utility remains to be defined. Our findings are hypothesis-generating and highlight the need for further research to determine whether [¹⁸F]PARPi PET adds value over [^68^Ga]PSMA PET or predicts response to PARP inhibitor therapy. Future studies should expand patient cohorts, correlate imaging findings with clinical and therapeutic outcomes, and explore the utility of combinatorial PSMA/PARPi theranostic approaches to better define the role of [¹⁸F]PARPi PET in clinical practice.

## Conclusion

The findings of this study demonstrate a considerable uptake of [^18^F]PARPi in mCRPC patients, particularly in osseous metastatic lesions, and reveal a relationship between [^18^F]PARPi- and [^68^Ga]PSMA uptake. These results suggest the potential of [^18^F]PARPi as a novel diagnostic imaging tool for mCRPC. The ability of [^18^F]PARPi PET to detect lesions with elevated PARP1 expression provides an option for further lesion characterization, independent of PSA levels or HRR pathway mutational status. The reported association between [^18^F]PARPi- and [^68^Ga]PSMA uptake suggests that PARPi PET imaging might add a value complementary to PSMA-based diagnostics, deeper insights into tumor biology and enabling more precise patient management.

In the future, the use of PARPi PET imaging, might aid personalized treatment strategies and be a helpful tool in clinical decision-making. Additionally, patients who would benefit from further treatment identification, targeting both receptor pathways, might be identified in the future; nevertheless, clinical trials evaluating the safety and utility of combined use of these therapeutic agents are still needed. Further studies will aim to validate the predictive value of [^18^F]PARPi uptake for therapeutic outcomes, assessing its ability to identify responders and non-responders to PARP inhibitor treatment strategies; thereby improving therapeutic outcomes in a patient cohort with limited treatment options. Ongoing research will be required to investigate this tracer in larger prospective cohorts.

## Data Availability

The datasets generated and/or analyzed in this study are available from the corresponding author upon reasonable request.
